# ‘SIRT8’ expressed in thyroid cancer is actually SIRT7

**DOI:** 10.1038/sj.bjc.6600635

**Published:** 2002-11-26

**Authors:** R Frye

**Affiliations:** Pittsburgh VA Med Center, Pathology (132L-U), University Drive C, Pittsburgh, Pennsylvania, PA 15240, USA

## Abstract

*British Journal of Cancer* (2002) **87**, 1479–1479. doi:10.1038/sj.bjc.6600635 www.bjcancer.com

© 2002 Cancer Research UK

## 

de Nigris *et al* (2002) claim to have isolated a cDNA prepared from thyroid papillary carcinoma that represents a novel gene of the silent information regulator (SIR) protein family, which the authors designated SIRT8. In fact, examination of their data reveals that the cDNA is derived from the previously characterised SIRT7 sequence (Frye, 2000). Moreover, the first 300 bases at the 5′ end of the supposedly novel cDNA depicted in Figure 1B of their article is none other than a sequence of the cloning vector, pBlue scriptIISK. The bases from 323 onward appear to be the SIRT7 sequence with some scattered sequencing errors. The small block of predicted amino-acids around codon 221 that diverge from SIRT7 can be accounted for by two 1-base frame shifts that are probably sequence artifacts. The ‘SIRT8’ sequence WYTGAGISTAASIPDYRGP is actually identical to the SIRT7 deposited in GenBank (NM_016538) which the authors incorrectly reproduced in Figure 1C of their article.

One must further doubt the validity of the authors' statement on ‘the homology of SIR-T8 with the telomerase proteins' because, while both types of protein have been implicated in ageing, they do not share structural homology or similar molecular functions. It is regrettable that neither the authors nor the editorial review process identified these errors.
